# Status and influencing factors of dual health literacy in modern medicine and traditional Chinese medicine among Chinese residents

**DOI:** 10.3389/fpubh.2025.1525282

**Published:** 2025-05-27

**Authors:** Fenfang Mi, Wenkai Zhou, Lingzhi Wang, Fang Yuan, Min Qian, Hongxia Zhang, Ningjun Xu

**Affiliations:** ^1^College of Humanities and Management, Zhejiang Chinese Medical University, Hangzhou, China; ^2^Department of Epidemiology and Biostatistics, School of Public Health, Zhejiang University School of Medicine, Hangzhou, China

**Keywords:** dual health literacy, traditional Chinese medicine, modern medicine, influencing factors, Chinese residents

## Abstract

**Background:**

Dual Health Literacy (DHL), integrating modern and traditional Chinese medicine (TCM), is crucial for health management in China. However, many struggle with both systems, causing fragmented decisions and poor outcomes. Most studies address only one system, overlooking their interplay. This study bridges the gap by assessing DHL and its key influencing factors to support integrated healthcare.

**Methods:**

Based on standardized 2017 questionnaires, this survey assessed health literacy based on modern medicine (HL) and traditional Chinese medicine (TCM-HL) among Chinese residents aged 15 to 69, using sampling via an online Sojump questionnaire. Group differences were assessed using the Mann–Whitney and Kruskal-Wallis test for continuous variables, and the chi-square test for categorical variables. Multivariate logistic regression was employed to identify factors associated with HL, TCM-HL, and DHL.

**Results:**

A total of 605 participants (median age: 23.0 years, IQR: 20–45) were surveyed, with the majority being female (69.4%), rural residents (59.8%), or holding junior college or undergraduate education (68.4%). Standard attainment rates were 27.1% for HL, 10.9% for TCM-HL, and 6.8% for DHL, with a strong correlation between HL and TCM-HL (r = 0.81). The lowest attainment was observed in basic medical literacy (13.2%), health skills (15.0%), chronic disease prevention & control (16%) within HL, and healthy lifestyle (0%) and appropriate methods of public health within TCM-HL (3.5%). DHL was higher in suburban and urban areas than in rural areas (13.3 and 9.4% vs. 4.7%; χ^2^ = 6.453, *p* = 0.04). Urban residence (AOR = 1.60, 95% CI: 1.09–2.34, *p* = 0.016), higher education level (AOR = 1.64, 95% CI: 1.22–2.21, *p* = 0.001) and health insurance coverage (AOR = 2.74, 95% CI: 1.12–6.68, *p* = 0.027) were significantly associated with higher HL attainment. Higher education level (AOR = 1.78, 95% CI: 1.33–2.37, *p* < 0.001) was significantly associated with TCM-HL attainment.

**Conclusion:**

Given the strong correlation between HL and TCM-HL, promoting the integrated concept of DHL is essential. The low DHL level underscores the need for targeted support, particularly for rural, less educated and uninsured residents. Efforts should enhance both modern and TCM health strategies, emphasizing health skills, chronic disease prevention and basic medical literacy in HL and healthy lifestyle and appropriate public health approaches in TCM-HL.

## 1 Introduction

Health literacy (HL), rooted in modern medicine, refers to an individual’s ability to obtain, understand, evaluate, and apply health information to make informed decisions regarding health maintenance, disease prevention, and treatment (1, 2). In parallel, traditional Chinese medicine health literacy (TCM-HL) reflects one’s ability to comprehend and utilize TCM-based health information, including disease etiology, treatment approaches, and preventive practices (3). Both HL and TCM-HL are vital in shaping health behaviors and outcomes, and essential to navigating China’s dual healthcare system, where modern medicine and TCM serve as complementary pillars. However, many individuals experience low HL or TCM-HL, leading to fragmented decision-making, poor treatment adherence, and reduced health outcomes, especially in the context of chronic diseases and aging populations. These issues highlight a problem gap, where insufficient literacy in either system compromises effective self-care and informed healthcare utilization.

Recognizing the importance of coordinated care, national policies such as *Healthy China 2030* (4) and the White Paper *Traditional Chinese Medicine in China* (5) have emphasized the equal importance of modern and traditional medical systems. This has created an urgent need to empower the population with the capacity to understand and apply health knowledge from both systems (6). In response, we introduce the concept of “dual health literacy” (DHL), the integrated ability to access, interpret, and use health-related information from both modern medicine and TCM. DHL is particularly relevant to preventive care, chronic disease self-management, and health promotion, where combining insights from both systems may improve outcomes and enhance patient engagement.

Although the importance of HL and TCM-HL is well-recognized, most existing studies examine them separately, focusing primarily on prevalence (7, 8), sociodemographic predictors (9), and associations with health outcomes (10, 11), few have explored their integration or synergistic effects, highlighting a clear research gap in the empirical assessment of DHL as a unified construct. To address this gap, this study aims to assess the current status of DHL among Chinese residents, identify key sociodemographic factors influencing it, and provide evidence to support targeted health education and policy. This study supports the development of integrated health literacy strategies and may offer insights for countries seeking to harmonize modern and traditional medical systems.

## 2 Materials and methods

### 2.1 Study area and period

This study was conducted across multiple regions in China to ensure diverse population representation. Data were collected in January 2023 through an online survey distributed via the Sojump platform, a widely used digital questionnaire tool in China.

### 2.2 Study design

A cross-sectional study was conducted to assess DHL by integrating measurements of HL and TCM-HL among Chinese residents.

### 2.3 Study population

The study population comprised Chinese residents aged 15 to 69 years.

### 2.4 Sample size determination

The final sample size was determined based on the number of valid responses received. The calculation of the sample size N in this study is as follows:


$$N=\frac{Z^{2}\times \sigma^{2}}{d^{2}}$$

In this context, Z represents the z-score corresponding to a 95% confidence level, which is 1.96; σ stands for the standard deviation, set to 0.8; d represents the margin of error, set to 0.07. Based on these values, the required sample size was calculated to be 502. Considering factors such as the non-response rate, we increased the sample size by approximately 20% to 600. A total of 605 fully completed and internally consistent questionnaires were included.

### 2.5 Sampling technique and procedure

Through the Sojump platform, participants were invited either orally or via WeChat communication. After receiving the invitation, participants gave informed consent and completed the questionnaire online. Only responses that were complete and met quality standards were included in the final analysis.

### 2.6 Data collection tools and procedure

Two validated instruments were used to assess HL and TCM-HL:

The “National Residents’ Health Literacy Monitoring Questionnaire (Version 2017)” includes 56 items across three core dimensions: basic knowledge and concepts, healthy lifestyle and behavior, and health skills (12). The “Chinese Citizens’ TCM Health Literacy Questionnaire (Version 2017)” consists of 37 items covering five dimensions: basic concepts of TCM, public health methods, TCM-based healthy lifestyle, TCM cultural knowledge, and the ability to understand TCM-related information (13).

Both questionnaires were developed by national authorities and have demonstrated good reliability and validity. Despite being introduced in 2017, they remain the most widely used and authoritative instruments in the field of health literacy in China.

### 2.7 Operational definitions and measurements

For HL, correct answers to True/False and Single Choice items were awarded 1 point, and correct answers to Multiple Choice items were awarded 2 points. A score ≥58 (80% of the total) was considered adequate HL. For TCM-HL, correct answers to True/False and Single Choice items were given 2 points, and Multiple Choice items 4 points. A score ≥80 (80% of the total) was considered adequate TCM-HL. Participants who met both criteria were classified as having Dual Health Literacy (DHL).

### 2.8 Data quality management

We pretested the TCM-HL questionnaire among 60 Chinese residents in the pilot investigation, demonstrating acceptable reliability (Cronbach’s ɑ = 0.768 for TCM lifestyle dimension; all dimensions >0.6). Exploratory factor analysis showed good structural validity (>50% variance explained). Spearman’s correlation analysis confirmed strong associations between specific dimensions and the total score (*ρ* = 0.705–0.827, all *p* < 0.001), demonstrating adequate content validity. To ensure data quality, questionnaires were excluded if they met any of the following conditions: completion time <120 s or >1,800 s, duplicate IP addresses, incomplete responses, or internal inconsistencies. A pilot test was conducted with a small sample to ensure clarity, comprehensibility, and content validity prior to full deployment.

### 2.9 Data processing and analysis

Descriptive statistics were used to analyze the basic demographic characteristics of the participants. Prior to conducting inferential analyses, the assumptions of normality and homogeneity of variance were assessed. Normality was tested using the Shapiro–Wilk test, while Levene’s test was applied to evaluate homogeneity of variance. Since the data violated the assumptions of normality (all *p* < 0.05) and homogeneity of variance (all *p* < 0.05), non-parametric tests were used throughout. Continuous variables were expressed as medians and interquartile ranges (M, IQR). Group differences were assessed using the Mann–Whitney U test and Kruskal-Wallis H test, and correlations were analyzed using Spearman’s rank correlation. Categorical data were analyzed using the chi-square test. Multivariate logistic regression was employed to identify factors associated with HL, TCM-HL, and DHL. Diagnostic tests including variance inflation factors (VIFs) were performed to check for multicollinearity in each model. Additionally, the Hosmer-Lemeshow test was used to evaluate model fitness. All data cleaning and statistical analyses were performed using SPSS version 22.0.

### 2.10 Ethical considerations

The study protocol was reviewed and approved by the Ethics Committee of Zhejiang Chinese Medical University. All participants provided informed consent before participation.

## 3 Results

### 3.1 Sociodemographic characteristics of participants

A total of 605 participants were included in the study, with a median age of 23.0 (IQR: 20–45) years. Among them, 69.4% were female. The majority resided in rural areas (59.8%). Most participants were of Han ethnicity (95.5%). Regarding educational attainment, 68.4% had completed junior college or undergraduate education. Most participants (91.9%) were insured under a medical insurance scheme (Table 1).

**Table 1:** Basic information of respondents (*N* = 605).

Variate		*N* (%)
Gender	Male	185 (30.6)
	Female	420 (69.4)
Age (M, IQR)		23.0 (20–45)
Residence	Rural area	362 (59.8)
	Urban city	213 (35.2)
	Suburban	30 (5.0)
Ethnic	Han	578 (95.5)
	non-Han ethnic	27 (4.5)
Education level	Primary school and below	19 (3.1)
	Junior high school	66 (10.9)
	Senior high school/technical secondary	74 (12.2)
	Junior college /undergraduate	414 (68.4)
	Graduate and above	32 (5.3)
Personal monthly income	<2,000	303 (50.1)
	2,000–9,999	240 (39.7)
	≥10,000	62 (10.2)
Medical insurance	Uninsured	49 (8.1)
	Insured	556 (91.9)

### 3.2 Levels of HL, TCM-HL, and DHL

**Figure 1:**
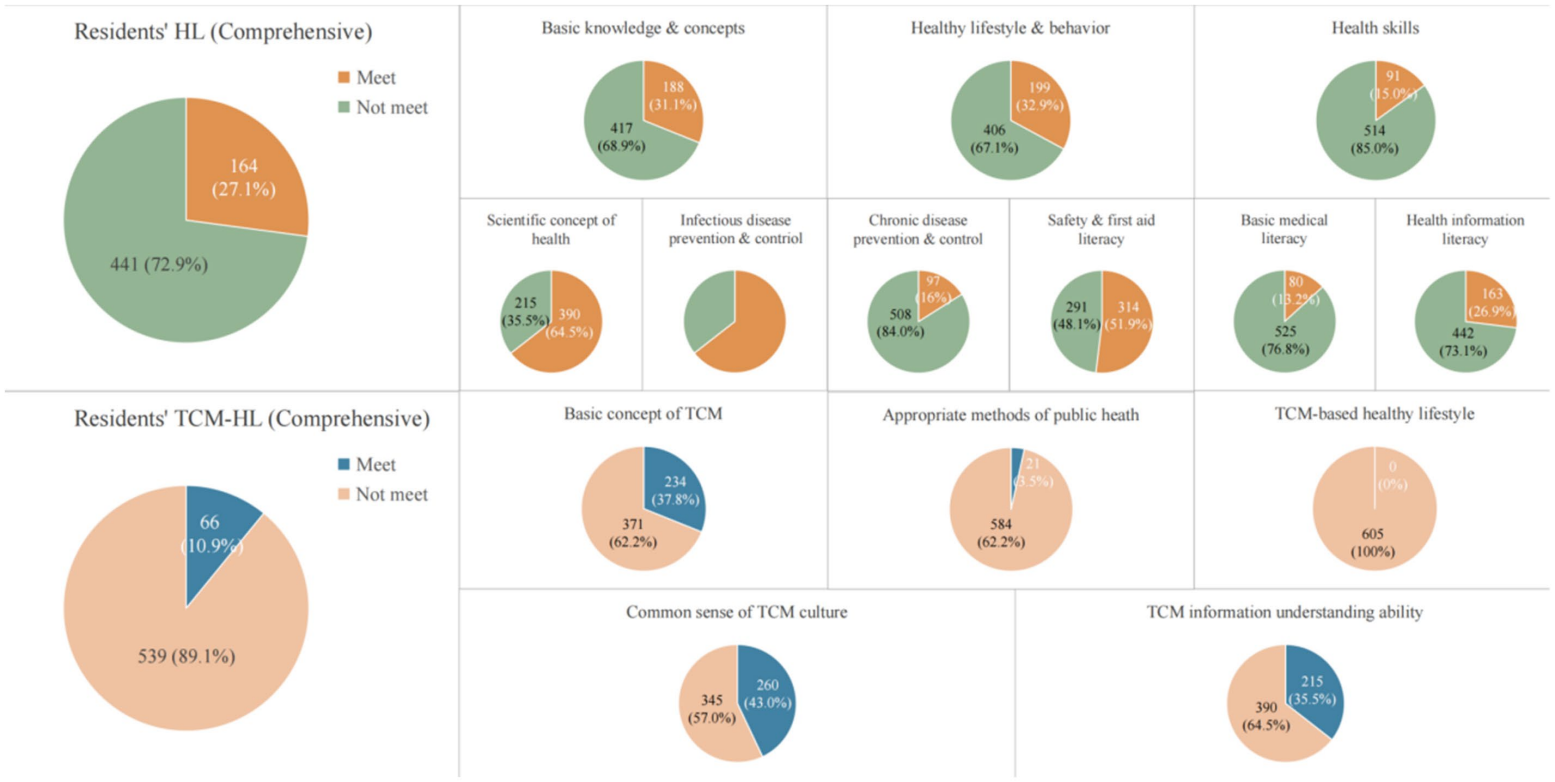
Residents’ HL and TCM-HL standard-reaching rate in difference dimensions.

The median comprehensive HL score among residents was 53 (IQR: 44–59), with 27.1% reaching the standard. Among the three HL aspects, the highest standard-reaching rate was observed in healthy lifestyle and behavior (32.9%), followed by basic knowledge and concepts (31.1%), and health skills (15.0%). The median comprehensive TCM-HL score was 68 (IQR: 52–76), with only 10.9% meeting the standard. Among the five dimensions, the highest standard-reaching rate was for common sense of TCM culture (43.0%), followed by TCM information understanding ability (35.5%) and basic TCM concepts (38.7%). In contrast, almost none met the standard in TCM healthy lifestyle (0%) and appropriate public health methods in TCM (3.5%) (Figure 1; Supplementary Table 1).

Females had significantly higher HL (M = 54.0, IQR = 47.0–59.0, U = 32986.5, *p* = 0.003) and TCM-HL scores (M = 68.0, IQR = 56.0–76.0, U = 33746.5, *p* = 0.01) than males (50.0, 34.5–58.5 for HL; 64.0, 37.0–76.0 for TCM-HL). HL scores differed significantly across age groups (H = 5.82, *p* = 0.016), with the <30 group scoring highest (54.0, 46.0–59.0), while TCM-HL did not show significant differences among age groups (*p* = 0.463). No significant differences were found between Han and non-Han ethnic groups in HL (*p* = 0.933; Han: 53.0, 44.0–59.0; non-Han: 55.0, 39.0–59.0) or TCM-HL (*p* = 0.665; Han: 68.0, 52.0–76.0; non-Han: 70.0, 44.0–76.0). Place of residence was associated with both HL (H = 9.02, *p* = 0.011) and TCM-HL (H = 12.57, *p* = 0.002), with urban residents scoring higher (HL: 54.0, 43.5–60.0; TCM-HL: 70.0, 58.0–76.0) than rural residents (HL: 52.0, 43.75–58.0; TCM-HL: 64.0, 50.0–74.0). Education level showed significant associations with HL (H = 39.54, *p* < 0.0001) and TCM-HL (H = 34.131, *p* < 0.0001), favoring those with higher education (Graduate and above: HL: 58.5, 37.25–63.0; TCM-HL: 69.0, 52.5–78.0; primary or below: HL: 46.0, 25.0–54.0; TCM-HL: 56.0, 30.0–68.0). No significant differences were found across income levels for HL (*p* = 0.408) or TCM-HL (*p* = 0.894) (Table 2).

**Table 2:** Scores of residents with different social demographic characteristics in HL and TCM-HL.

Variables	*n*	Residents’ HL (comprehensive)			Residents’ TCM-HL (comprehensive)		
		Median, IQR	Test statistic	*p*-value	Median, IQR	Test statistic	*p*-value
Total (M ± S)	605	48.6 ± 15.1	/	/	61.5 ± 19.6	/	/
Gender							
Male	185	50 (34.5–58.5)	32986.5^#^	0.003	64.0 (37.0–76.0)	33746.5^#^	0.01
Female	420	54 (47.0–59.0)			68.0 (56.0–76.0)		
Different ages							
<30	375	54.0 (46.0–59.0)	5.8^*^	0.016	68.0 (54.0–76.0)	0.5^*^	0.463
30–59	223	52.0 (43.0–59.0)			68.0 (50.0–76.0)		
≥60	7	38.0 (22.0–49.0)			44.0 (38.0–76.0)		
Different ethnics							
Han	578	53.0 (44.0–59.0)	7728.5^#^	0.933	68.0 (52.0–76.0)	7419.0^#^	0.665
non-Han ethnic	27	55.0 (39.0–59.0)			70.0 (44.0–76.0)		
Different residence							
Rural	362	52.0 (43.75–58.0)	9.0^*^	0.011	64.0 (50.0–74.0)	12.6^*^	0.002
Urban	213	54.0 (43.5–60.0)			70.0 (58.0–76.0)		
Suburban	30	49.5 (58.0–61.0)			71.0 (52.0–80.0)		
Different levels of education							
Primary school and below	19	46.0 (25.0–54.0)	39.5^*^	<0.0001^#^	56.0 (30.0–68.0)	34.1^*^	<0.0001
Junior high school	66	45.5 (36.0–52.0)			55.0 (35.0–70.0)		
Senior high School/technical Secondary	74	51.5 (38.75–58.0)			66.0 (46.5–74.0)		
Junior college/undergraduate	414	54.0 (48.0–59.0)			68.0 (58.0–76.0)		
Graduate and above	32	58.5 (37.25–63.0)			69.0 (52.5–78.0)		
Personal monthly income							
<2,000	303	54.0 (44.0–59.0)	1.8^*^	0.408	66.0 (54.0–74.0)	0.2^*^	0.894
2,000–10,000	240	52.0 (44.0–59.0)			68.0 (50.0–76.0)		
>10,000	62	53.0 (43.0–62.25)			66.0 (53.5–76.5)		

^#^Mann–Whitney Test, and the test statistic was the Mann–Whitney U value. ^*^Kruskal–Wallis H test, and the test statistic was the chi-square value (H).

### 3.3 Standard-reaching rate in HL, TCM-HL, and DHL

**Figure 2:**
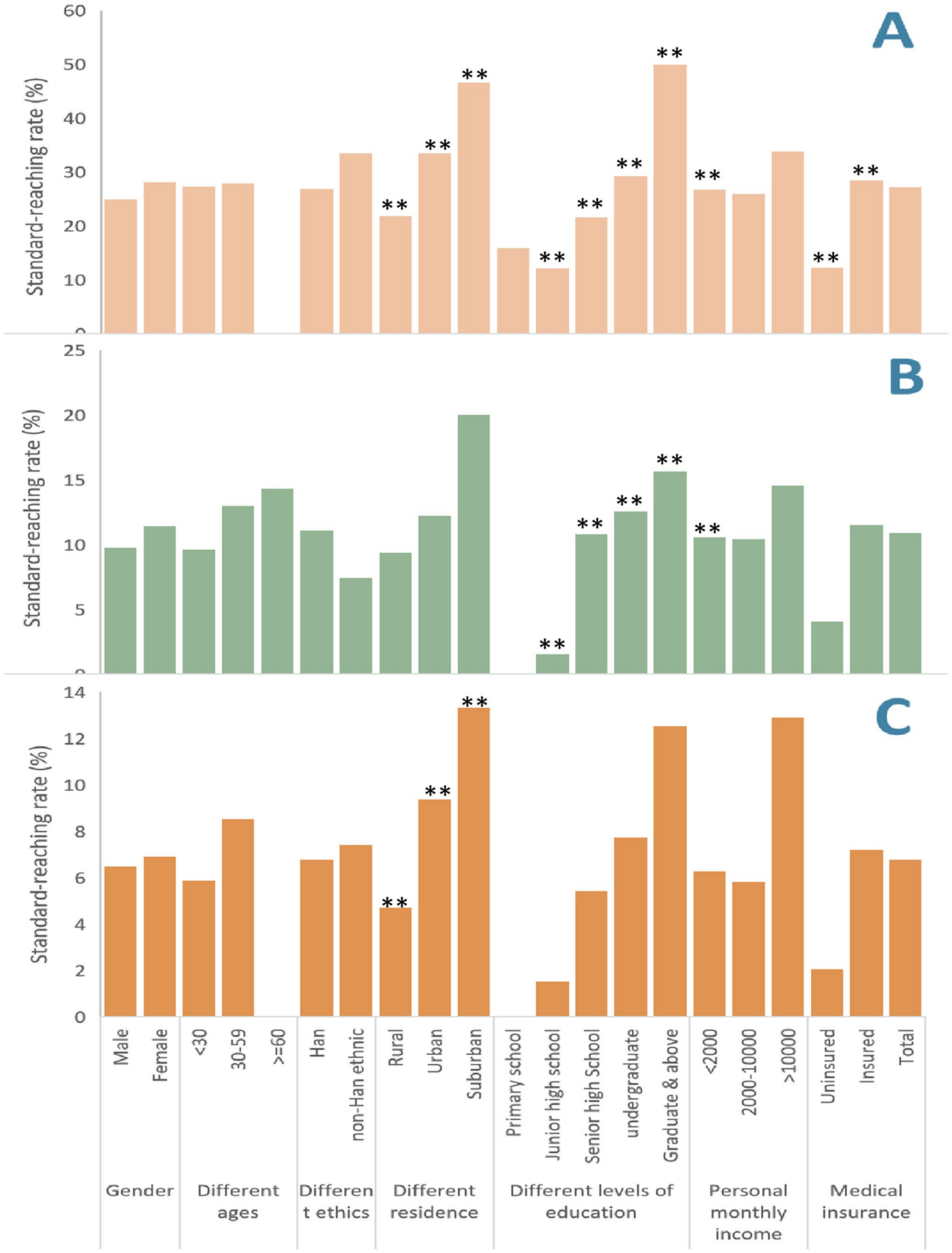
RResident’s HL and TCW-HL standard-reaching rate in different dimensions. **(A)** Residence’s HL standard-reaching rate in different dimensions; **(B)** Residence’s TCM-HL standard-reaching rate in different dimensions; **(C)** Residence’s DHL standard-reaching rate in different dimensions. **denotes *p* <0.01.

No significant gender differences in standard-reaching rate were observed in HL (male: 24.9%, female: 28.1%, *p* = 0.408), TCM-HL (9.7% vs. 11.4%, *p* = 0.533), or DHL (6.5% vs. 6.9%, *p* = 0.850). Although HL appeared slightly higher in younger age groups (<30: 27.2%, 30–59: 27.8%), the differences were not statistically significant (*p* = 0.106). Similar nonsignificant trends were seen in TCM-HL and DHL across age groups. HL was highest in suburban (46.7%) and urban (33.3%) areas, and lowest in rural areas (21.8%) (χ^2^ = 14.57, *p* = 0.001). DHL showed a similar trend (suburban: 13.3%, urban: 9.4%, rural: 4.7%) (χ^2^ = 6.45, *p* = 0.04). HL ranged from 15.8% in those with primary education or below to 50.0% among those with graduate-level education (χ^2^ = 19.82, *p* = 0.001). TCM-HL followed the same trend, from 0% in the lowest group to 15.6% in the highest (χ^2^ = 15.24, *p* = 0.004). The difference in DHL by education was not statistically significant (χ^2^ = 8.96, *p* = 0.062), but showed an upward trend (0 to 12.5%). Medical insurance status was significantly related to HL (χ^2^ = 6.90, *p* = 0.009), with insured participants showing higher rates (28.4%) than uninsured (12.2%). No significant differences in any literacy measure were observed across income groups (Figure 2; Supplementary Table 2).

### 3.4 Factors associated with HL and TCM-HL

The univariate logistic regression analysis indicated that higher education level (OR = 1.65, 95% CI: 1.28–2.13, *p* < 0.001), urban residence (OR = 1.93, 95% CI: 1.34–2.77, *p* < 0.001), higher monthly income (OR = 2.74, 95% CI: 1.10–6.83, *p* = 0.030), and having health insurance (OR = 2.85, 95% CI: 1.19–6.82, *p* = 0.019) were significantly associated with HL standard-reaching rates. Similarly, for TCM-HL standard-reaching rates, higher education level (OR = 1.86, 95% CI: 1.24–2.79, *p* = 0.003) and higher monthly income (OR = 3.63, 95% CI: 1.30–10.11, *p* = 0.014) showed significant associations. All variance inflation factors (VIFs) of variables in multivariate models were below 5 (range: 1.039–2.541), indicating no concerning multicollinearity among the included variables. Multivariate logistic regression analysis showed that urban residence (AOR = 1.60, 95% CI: 1.09–2.34, *p* = 0.016), higher education (AOR = 1.64, 95% CI: 1.22–2.21, *p* = 0.001), and health insurance coverage (AOR = 2.74, 95% CI: 1.12–6.68, *p* = 0.027) were significantly associated with higher odds of HL standard attainment, while gender, age, and income level were not. Similarly, higher education (AOR = 1.78, 95% CI: 1.33–2.37, *p* < 0.001) was significantly associated with TCM-HL attainment. Gender, income and insurance status showed no significant associations (Table 3). The Hosmer-Lemeshow test for the HL model indicated a good model fit (χ^2^ = 1.422, *p* = 0.994). Similarly, the TCM-HL model also demonstrated a good fit (χ^2^ = 9.210, *p* = 0.325).

**Table 3:** Multivariate and univariate analyses of factors influencing HL and TCM-HL standard-reaching rates.

Types of literacy	Variables	Uni-variate analysis			Multivariate analysis		
		B	*p*-value	OR (95% CI)	B	*p*-value	AOR (95% CI)
Residents’ HL (comprehensive score)	Gender (female vs. male)	0.17	0.410	1.18 (0.80–1.75)	0.05	0.834	1.05 (0.69–1.59)
	Age (30–60 vs. <30)	0.03	0.873	1.03 (0.71–1.49)	0.47	0.144	1.60 (0.85–2.99)
	Different places of residence (urban vs. rural)	0.66	0.000	1.93 (1.34–2.77)	0.47	0.016	1.60 (1.09–2.34)
	Educational level (grade)	0.50	0.000	1.65 (1.28–2.13)	0.50	0.001	1.64 (1.22–2.21)
	Monthly income (2,000–3,999 vs. <2000)	0.05	0.874	1.05 (0.58–1.91)	0.06	0.851	1.06 (0.56–2.02)
	Monthly income (4,000–5,999 vs. <2000)	−0.13	0.624	0.88 (0.52–1.48)	−0.08	0.812	0.92 (0.48–1.78)
	Monthly income (6,000–7,999 vs. <2,000)	−0.02	0.941	0.98 (0.55–1.73)	−0.25	0.515	0.78 (0.37–1.66)
	Monthly income (8,000–9,999 vs. <2,000)	−0.03	0.941	0.97 (0.47–2.03)	−0.55	0.266	0.58 (0.22–1.52)
	Monthly income (>10,000 vs. <2,000)	1.01	0.030	2.74 (1.10–6.83)	0.32	0.567	1.38 (0.46–4.16)
	Health insurance (Yes vs. No)	1.05	0.019	2.85 (1.19–6.82)	1.01	0.027	2.74 (1.12–6.68)
Residents’ TCM-HL (Comprehensive sores)	Gender (Female vs. Male)	0.18	0.537	1.20 (0.68–2.12)	0.07	0.758	1.07 (0.71–1.61)
	Age (30–60 vs. <30)	0.34	0.197	1.41 (0.84–2.37)	0.54	0.084	1.72 (0.93–3.18)
	Educational level (grade)	0.62	0.003	1.86 (1.24–2.79)	0.58	0.000	1.78 (1.33–2.37)
	Monthly income (2,000–3,999 vs. <2,000)	−0.89	0.150	0.41 (0.12–1.38)	0.10	0.753	1.11 (0.59–2.09)
	Monthly income (4,000–5,999 vs. <2,000)	0.06	0.878	1.06 (0.51–2.19)	−0.11	0.752	0.90 (0.47–1.72)
	Monthly income (6,000–7,999 vs. <2,000)	0.36	0.338	1.43 (0.69–2.99)	−0.25	0.515	0.78 (0.37–1.64)
	Monthly income (8,000–9,999 vs. <2000)	−0.43	0.495	0.65 (0.19–2.23)	−0.46	0.344	0.63 (0.25–1.63)
	Monthly income (>10,000 vs. <2000)	1.29	0.014	3.63 (1.30–10.11)	0.35	0.527	1.42 (0.48–4.17)

Significant correlations were found between various dimensions of HL and TCM-HL, with a strong overall correlation between the two. For HL, the strongest correlation in HL was between basic knowledge & concepts and the comprehensive HL score (0.96), while the weakest was between health information literacy and infectious disease prevention literacy (0.53). For TCM-HL, the strongest correlation was between the basic concept of TCM and the comprehensive TCM-HL score (0.90), with the weakest being between TCM information understanding ability and appropriate methods of public health in TCM (0.52). Most notably, a strong overall correlation was found between HL and TCM-HL (0.81), with the weakest correlation observed between infectious disease prevention and control literacy in HL and information understanding ability in TCM-HL (0.42). These findings highlight the interconnection of different dimensions of HL and TCM-HL (Supplementary Table 3).

## 4 Discussion

### 4.1 Key findings

This study introduced the integrated concept of DHL among Chinese residents, and assessed the levels and influencing factors of HL, TCM-HL, and DHL. The median scores for HL and TCM-HL were 53 and 68, respectively, with 27.1% and 10.9% of participants attaining the corresponding standards; however, only 6.8% attained the standard for DHL.

Univariate analysis showed females, urban residents, and individuals with higher education scored significantly higher in both HL and TCM-HL, though gender differences should be interpreted cautiously due to the over-representation of females. Multivariate analysis identified urban residence, higher education, and medical insurance coverage as key predictors of higher HL and/or TCM-HL attainment. The findings revealed notable disparities across sociodemographic groups, highlighting the need for targeted health education interventions.

### 4.2 Interpretation and comparison with previous literature

The low attainment rates of HL (27.1%), TCM-HL (10.9%), and especially DHL (6.8%) highlight a significant gap in residents’ ability to engage with both medical systems effectively. These findings are in line with previous research indicating that both HL and TCM-HL levels in China remain relatively low. A national survey in 2023 reported a general HL attainment rate of approximately 29.70% (14), while a 2022 provincial study in Jiangsu found that TCM-HL was just 14.76% (15). And the markedly lower DHL rate compared to HL and TCM-HL suggests that possessing dual literacy is considerably more challenging than acquiring each type separately.

Meanwhile, identifying specific weaknesses in HL and TCM-HL is crucial for improving overall DHL. Regarding HL, the standard-reaching rates for basic medical literacy (13.2%), health skills (15%), and chronic disease prevention and control literacy (16%) were notably low. This aligns with findings from Anhui Province, where the lowest scores were also in health skills (28.21%), and medical literacy (25.71%) (16). The relatively lower rates in this study may be attributed to a smaller proportion of urban participants (35.2%), as urban residents tend to have better access to health information and services. Regarding TCM-HL, the standard-reaching rates for appropriate public health methods in TCM (3.5%) and TCM-based healthy lifestyles (0.0%) were extremely low. These results are broadly consistent with surveys in Liaoning (3.6%) (17) and Zhejiang (5.76%) (18) provinces. However, both studies reported comparatively higher literacy levels for TCM-based lifestyles than those observed here. This discrepancy may reflect regional differences in TCM promotion, cultural acceptance, or resource availability.

Multivariate analysis indicated that urban residence, higher education, and health insurance coverage were significantly associated with a higher HL level, whereas education level was the sole significant determinant of TCM-HL. Collectively, these factors play a critical role in shaping the overall DHL. Specifically, participants living in urban areas had 1.60 times higher odds (95% CI: 1.09–2.34) of attaining adequate HL compared to rural residents, while those with higher education had 1.64 times greater odds (95% CI: 1.22–2.21). Similar findings have been reported in both Chinese (7, 19) and international studies (20, 21), underscoring the role of residential context in shaping health literacy. The regional differences may stem from disparities in education, healthcare access, and socioeconomic factors between urban and rural areas. Besides, the strong association between higher education and both HL and TCM-HL is consistent with national surveys in China (22) and international research in developed countries such as the United States (23) and Australia (24), where individuals with tertiary education consistently report better HL outcomes. One possible explanation is that higher education improves individuals’ ability to access, understand, and apply health information. Additionally, access to health insurance was associated with significantly greater HL attainment (AOR = 2.74, 95% CI: 1.12–6.68), aligning with findings from prior studies that emphasize the role of healthcare access in promoting health literacy (25). This association may stem from health insurance enhancing access to healthcare services, increasing exposure to health information and resources, and ultimately enhancing HL. In contrast, TCM-HL was associated only with education level in this adjusted model (AOR = 1.78, 95% CI: 1.33–2.37), while other factors such as gender and age were not significant. This may reflect the more specialized nature of TCM knowledge and its stronger dependence on formal or targeted health education.

The low level of DHL in China cannot be attributed solely to demographic factors; rather, it reflects deeper issues related to knowledge systems, cultural perceptions, and resource distribution. First, modern and traditional medicine are often taught in isolation within the health education system, lacking integration that would help the public understand their complementary roles. This fragmented approach undermines both comprehension and public trust in a dual-track model of health management. Second, cultural stereotypes and cognitive biases further hinder the absorption of integrated health information. Many individuals hold fixed beliefs, such as “TCM is gentle but slow” or “Western medicine is effective but comes with side effects,” which affect health-related decision-making. Third, access to high-quality integrative health education resources remains highly uneven, particularly in rural and remote areas. Although digital resources are expanding, the digital divide continues to restrict access to reliable health information for many, especially those in under-served communities. These factors collectively impede the public’s understanding and application of integrated medical knowledge, contributing to the persistently low levels of DHL in China.

### 4.3 Implications for public health practice

The findings emphasize the urgent need to establish DHL as a unified framework for public health education in China. Collaboration between policymakers, healthcare providers, and community leaders will be crucial in promoting DHL and fostering a more health-literate society, enabling individuals to make informed health decisions and engage with both modern medicine and traditional health practices.

To enhance DHL, it is essential to address critical gaps in both HL and TCM-HL. For HL, focused efforts should improve health skills, chronic disease prevention, and basic medical knowledge. For TCM-HL, priority should be given to enhancing knowledge of TCM-based public health methods and healthy lifestyles, especially in regions with lower literacy levels.

Interventions should prioritize rural residents, individuals with lower education levels, and those without health insurance, as these groups typically exhibit lower literacy rates. Public health campaigns must be tailored to the specific needs of these populations, utilizing school-based programs, community outreach, and digital platforms to increase access to health information. Expanding health insurance coverage and integrating it with health education initiatives can empower individuals to make informed decisions by increasing access to both health services and knowledge.

A comprehensive strategy is essential. First, health education should integrate modern and traditional medicine by creating unified curricula and adopting collaborative teaching methods that emphasize their complementary roles. Second, cultural stereotypes and cognitive biases must be addressed through effective science communication, utilizing accessible language and real-life examples to reshape public perceptions of both systems. Third, addressing the resource gap, particularly in rural areas, is critical. Expanding digital platforms with integrated health content will ensure wider access to comprehensive health knowledge. Additionally, collaboration between community health centers, universities, and hospitals to provide both online and in-person training will further enhance health literacy.

These efforts will boost DHL, deepen the understanding of integrated health practices, and promote the adoption of a dual-track health management approach.

### 4.4 Strength and limitation

This study has several notable strengths. It employed two nationally recognized and widely used instruments—the “National Residents’ Health Literacy Monitoring Questionnaire” and the “Chinese Residents’ TCM Health Literacy Questionnaire (2017 version)”—both of which have demonstrated good reliability and validity. It also introduced the integrated concept of DHL, assessing both HL and TCM-HL simultaneously to better reflect the structure of China’s dual healthcare system.

However, this study has several limitations. First, the use of sampling may introduce selection bias and limit generalizability. To mitigate this, we recruited participants from multiple regions across China and applied strict quality control procedures, including exclusion of incomplete or inconsistent responses and time-based validity checks. Nevertheless, certain populations, such as older adults, individuals without internet access, or those in remote areas, may have been underrepresented. Future research should consider probability sampling or mixed-method approaches to improve representativeness. Second, the gender distribution was skewed, with 69.4% female participants, which may have influenced the observed gender differences in HL and TCM-HL. These comparisons should be interpreted cautiously, and future studies with more balanced gender representation are recommended.

## 5 Conclusion

Given the significant correlation and mutual reinforcement between HL and TCM-HL, promoting the integrated concept of DHL is both timely and essential. However, the overall level of DHL remains low, underscoring the need for targeted, equity-oriented interventions. Priority should be given to enhancing basic medical literacy, chronic disease prevention, and practical health skills within HL, as well as promoting healthy lifestyle practices and appropriate public health approaches within TCM-HL. Health education efforts should aim to integrate modern medicine and TCM, with a focus particularly for rural, less educated and uninsured residents. To achieve these goals, integrating medical education with cultural values, expanding digital platforms for under-served populations, promoting cross-sector collaboration, and coordinating resource allocation are essential.

## Data Availability

The raw data supporting the conclusions of this article will be made available by the authors, without undue reservation.
