# Supplementary material for: Status and influencing factors of dual health literacy in modern medicine and traditional Chinese medicine among Chinese residents
Source: Front Public Health. 2025 May 27;13:1525282. doi: 10.3389/fpubh.2025.1525282 (PMC12149185; doi:10.3389/fpubh.2025.1525282)
Supplement: Supplementary file 1 [file Table_1.docx]

**Supplementary Table 1.** Residents' HL, TCM-HL, scores in different dimensions and the standard-reaching rate.

| **Type of HL** | **Score** | **Standard-reaching number & rate (%)** |
| --- | --- | --- |
| Residents' HL (Comprehensive) (M，IQR) | 53(44-59) | 164(27.1) |
| Residents' HL (3 Aspects) |  |  |
| Basic knowledge & concepts (M，IQR) | 25(21-27) | 188(31.1) |
| Healthy lifestyle & behavior (M，IQR) | 17(14-20) | 199(32.9) |
| Health skills (M±S) | 9.7±3.9 | 91(15) |
| Residents' HL (6 Types of Questions) |  |  |
| Scientific concept of health (M±S) | 9.6±3 | 390(64.5) |
| Infectious disease prevention & control Literacy (M±S) | 5.1±2 | 157(26) |
| Chronic disease prevention & control literacy (M±S) | 8.5±3 | 97(16) |
| Safety & first aid literacy (M，IQR) | 13(10-14) | 314(51.9) |
| Basic medical literacy (M±S) | 9.1±3.3 | 80(13.2) |
| Health information literacy (M±S) | 4.9±2.1 | 163(26.9) |
|  |  |  |
| Residents' TCM-HL (Comprehensive) (M，IQR) | 68(52-76) | 66(10.9) |
| TCM-HL in 5 Dimensions |  |  |
| Basic concept of TCM (M±S) | 16.6±5.7 | 234(38.7) |
| Appropriate methods of public health in TCM (M±S) | 9.2±5.3 | 21(3.5) |
| TCM-based healthy lifestyle (M±S) | 23.5±6.3 | 0(0) |
| Common sense of TCM culture (M±S) | 7.2±3.2 | 260(43) |
| TCM information understanding ability (M±S) | 5±2.9 | 215(35.5) |
