# Supplementary material for: Status and influencing factors of dual health literacy in modern medicine and traditional Chinese medicine among Chinese residents
Source: Front Public Health. 2025 May 27;13:1525282. doi: 10.3389/fpubh.2025.1525282 (PMC12149185; doi:10.3389/fpubh.2025.1525282)
Supplement: Supplementary file 2 [file Table_2.docx]

**Supplementary Table 2.** Standard-reaching rate in HL, TCM-HL, and DHL among the residents with different social demographic characteristics

| **Variables** | **HL, N(%)** | **Chi-square statistic** | **p-value** |  | **TCM-HL, N(%)** | **Chi-square statistic** | **p-value** |  | **DHL, N(%)** | **Chi-square statistic** | **p-value** |
| --- | --- | --- | --- | --- | --- | --- | --- | --- | --- | --- | --- |
| Gender | | | | | | | | | | | |
| Male | 46(24.9) | 0.69 | 0.408 |  | 18(9.7) | 0.39 | 0.533 |  | 12(6.5) | 0.04 | 0.85 |
| Female | 118(28.1) |  |  |  | 48(11.4) |  |  |  | 29(6.9) |  |  |
| Different ages | | | | | | | | | | | |
| <30 | 102(27.2) | 4.48 | 0.106 |  | 36(9.6) | 1.72 | 0.424 |  | 22(5.9) | 2.49 | 0.287 |
| 30-59 | 62(27.8) |  |  |  | 29(13.0) |  |  |  | 19(8.5) |  |  |
| >=60 | 0(0) |  |  |  | 1(14.3) |  |  |  | 0(0) |  |  |
| Different ethics | | | | | | | | | | | |
| Han | 155(26.8) | 0.53 | 0.466 |  | 64(11.1) | 0.40 | 0.53 |  | 39(6.8) | 0.02 | 0.895 |
| non-Han ethnic | 9(33.33) |  |  |  | 2(7.4) |  |  |  | 2(7.4) |  |  |
| Different residence | | | | | | | | | | | |
| Rural | 79(21.8)** | 14.57 | 0.001 |  | 34(9.4) | 3.36 | 0.186 |  | 17(4.7)* | 6.45 | 0.04 |
| Urban | 71(33.3)** |  |  |  | 26(12.2) |  |  |  | 20(9.4)* |  |  |
| Suburban | 14(46.7)** |  |  |  | 6(20.0) |  |  |  | 4(13.3)* |  |  |
| Different levels of education | | | | | | | | | | | |
| Primary school & below | 3(15.8)** | 19.82 | 0.001 |  | 0(0)** | 15.24 | 0.004 |  | 0(0) | 8.96 | 0.062 |
| Junior high school | 8(12.1)** |  |  |  | 1(1.5)** |  |  |  | 1(1.5) |  |  |
| Senior high School | 16(21.6)** |  |  |  | 8(10.8)** |  |  |  | 4(5.4) |  |  |
| Undergraduate | 121(29.3)** |  |  |  | 52(12.6)** |  |  |  | 32(7.7) |  |  |
| Graduate & above | 16(50.0)** |  |  |  | 5(15.6)** |  |  |  | 4(12.5) |  |  |
| Personal monthly income | | | | | | | | | | | |
| <2000 | 81(26.7) | 1.59 | 0.451 |  | 32(10.6) | 0.86 | 0.651 |  | 19(6.3) | 3.44 | 0.179 |
| 2000-10000 | 62(25.8) |  |  |  | 25(10.4) |  |  |  | 14(5.8) |  |  |
| >10000 | 21(33.9) |  |  |  | 9(14.5) |  |  |  | 8(12.9) |  |  |
| Medical insurance | | | | | | | | | | | |
| Uninsured | 6(12.2)** | 6.90 | 0.009 |  | 2(4.1) | 3.21 | 0.073 |  | 1(2.0) | 2.51 | 0.113 |
| Insured | 158(28.4)** |  |  |  | 64(11.5) |  |  |  | 40(7.2) |  |  |
| Total | 164(27.1)** | / | / |  | 66(10.9) | / | / |  | 41(6.8) | / | / |

*: P<0.05; **: P<0.01.
