# Supplementary material for: Status and influencing factors of dual health literacy in modern medicine and traditional Chinese medicine among Chinese residents
Source: Front Public Health. 2025 May 27;13:1525282. doi: 10.3389/fpubh.2025.1525282 (PMC12149185; doi:10.3389/fpubh.2025.1525282)
Supplement: Supplementary file 3 [file Table_3.docx]

**Supplementary Table 3.** Residents' HL & TCM-HL and the correlation of different dimensions

| **Type of HL** | | **Basic knowledge & concept** | **Healthy lifestyle & behavior** | **Health skills** | **Scientific health concept** | **Infectious disease prevention & control literacy** | **Chronic disease prevention & control literacy** | **Safety & first aid literacy** | **Basic medical literacy** | **Health information literacy** | **Comprehensive literacy** |  | **TCM Basic knowledge & concepts** | **Appropriate methods of public health in TCM** | **TCMbased healthy lifestyle** | **Common sense of TCM culture** | **TCM information understanding ability** | **Comprehensive scores** |
| --- | --- | --- | --- | --- | --- | --- | --- | --- | --- | --- | --- | --- | --- | --- | --- | --- | --- | --- |
| Residents' HL | Basic knowledge & concept | 1.00 | 0.83 | 0.80 | 0.86 | 0.75 | 0.85 | 0.89 | 0.80 | 0.74 | **0.96** |  | 0.71 | 0.52 | 0.70 | 0.68 | 0.56 | 0.77 |
|  | Healthy lifestyle & behavior | 0.83 | 1.00 | 0.78 | 0.81 | 0.73 | 0.86 | 0.83 | 0.88 | 0.67 | 0.94 |  | 0.70 | 0.53 | 0.69 | 0.63 | 0.57 | 0.75 |
|  | Health skill | 0.80 | 0.78 | 1.00 | 0.81 | 0.64 | 0.73 | 0.87 | 0.76 | 0.79 | 0.90 |  | 0.68 | 0.54 | 0.68 | 0.67 | 0.58 | 0.76 |
|  | Scientific concept of health | 0.86 | 0.81 | 0.81 | 1.00 | 0.64 | 0.74 | 0.79 | 0.72 | 0.65 | 0.89 |  | 0.67 | 0.49 | 0.65 | 0.65 | 0.50 | 0.71 |
|  | Infectious disease prevention & control literacy | 0.75 | 0.73 | 0.64 | 0.64 | 1.00 | 0.59 | 0.66 | 0.63 | **0.53** | 0.76 |  | 0.54 | 0.47 | 0.56 | 0.58 | 0.42 | 0.62 |
|  | Chronic disease prevention literacy | 0.85 | 0.86 | 0.73 | 0.74 | 0.59 | 1.00 | 0.79 | 0.71 | 0.66 | 0.88 |  | 0.66 | 0.47 | 0.66 | 0.61 | 0.55 | 0.71 |
|  | Safety & first aid literacy | 0.89 | 0.83 | 0.87 | 0.79 | 0.66 | 0.79 | 1.00 | 0.76 | 0.66 | 0.93 |  | 0.70 | 0.51 | 0.68 | 0.66 | 0.58 | 0.75 |
|  | Basic medical literacy | 0.80 | 0.88 | 0.76 | 0.72 | 0.63 | 0.71 | 0.76 | 1.00 | 0.61 | 0.88 |  | 0.66 | 0.51 | 0.64 | 0.59 | 0.54 | 0.71 |
|  | Health information literacy | 0.74 | 0.67 | 0.79 | 0.65 | 0.53 | 0.66 | 0.66 | 0.61 | 1.00 | 0.78 |  | 0.61 | 0.47 | 0.59 | 0.56 | 0.52 | 0.66 |
|  | Comprehensive scores | 0.96 | 0.94 | 0.90 | 0.89 | 0.76 | 0.88 | 0.93 | 0.88 | 0.78 | 1.00 |  | 0.75 | 0.57 | 0.74 | 0.71 | 0.61 | 0.81 |
|  |  |  |  |  |  |  |  |  |  |  |  |  |  |  |  |  |  |  |
| Residents' TCM-HL | Basic concept of TCM | 0.71 | 0.70 | 0.68 | 0.67 | 0.54 | 0.66 | 0.70 | 0.66 | 0.61 | 0.75 |  | 1.00 | 0.60 | 0.76 | 0.66 | 0.63 | **0.90** |
|  | Appropriate methods of public health in TCM | 0.52 | 0.53 | 0.54 | 0.49 | 0.47 | 0.47 | 0.51 | 0.51 | 0.47 | 0.57 |  | 0.60 | 1.00 | 0.57 | 0.55 | 0.52 | 0.79 |
